# A novel hybrid peptide composed of LfcinB6 and KR-12-a4 with enhanced antimicrobial, anti-inflammatory and anti-biofilm activities

**DOI:** 10.1038/s41598-022-08247-4

**Published:** 2022-03-14

**Authors:** Chelladurai Ajish, Sungtae Yang, S. Dinesh Kumar, Eun Young Kim, Hye Jung Min, Chul Won Lee, Sung-Heui Shin, Song Yub Shin

**Affiliations:** 1 Department of Cellular and Molecular Medicine, Graduate School of Biomedical Science, School of Medicine, Chosun University, Gwangju, 61452 Republic of Korea; 2 Department of Microbiology, School of Medicine, Chosun University, Gwangju, 61452 Republic of Korea; 3 Department of Cosmetic Science, Gwangju Women’s University, Gwangju, 62396 Republic of Korea; 4 Department of Chemistry, Chonnam National University, Gwangju, 61186 Republic of Korea

**Keywords:** Biochemistry, Biophysics, Drug discovery

## Abstract

Hybridizing two known antimicrobial peptides (AMPs) is a simple and effective strategy for designing antimicrobial agents with enhanced cell selectivity against bacterial cells. Here, we generated a hybrid peptide Lf-KR in which LfcinB6 and KR-12-a4 were linked with a Pro hinge to obtain a novel AMP with potent antimicrobial, anti-inflammatory, and anti-biofilm activities. Lf-KR exerted superior cell selectivity for bacterial cells over sheep red blood cells. Lf-KR showed broad-spectrum antimicrobial activities (MIC: 4–8 μM) against tested 12 bacterial strains and retained its antimicrobial activity in the presence of salts at physiological concentrations. Membrane depolarization and dye leakage assays showed that the enhanced antimicrobial activity of Lf-KR was due to increased permeabilization and depolarization of microbial membranes. Lf-KR significantly inhibited the expression and production of pro-inflammatory cytokines (nitric oxide and tumor necrosis factor‐α) in LPS-stimulated mouse macrophage RAW264.7 cells. In addition, Lf-KR showed a powerful eradication effect on preformed multidrug-resistant *Pseudomonas aeruginosa* (MDRPA) biofilms. We confirmed using confocal laser scanning microscopy that a large portion of the preformed MDRPA biofilm structure was perturbed by the addition of Lf-KR. Collectively, our results suggest that Lf-KR can be an antimicrobial, anti-inflammatory, and anti-biofilm candidate as a pharmaceutical agent.

## Introduction

The increasing emergence and dissemination of antibiotic resistance have become a global public health challenge^1^. Therefore, there is an urgent need to develop new antimicrobial agents to overcome this problem. In recent years, antimicrobial peptides (AMPs) have attracted considerable interest as promising therapeutic alternatives to conventional antibiotics because of their broad-spectrum antimicrobial activity, membrane-targeting antimicrobial mechanism, rapid killing, and infrequent development of drug resistance^2,3^. AMPs are an essential component of the innate immune system and are produced as the first line of defense by multicellular organisms^2,3^. Furthermore, they possess immunomodulatory properties such as leukocyte recruitment and suppression of harmful inflammation^3^. One strategy to design novel AMPs with enhanced antimicrobial activity while reducing cytotoxicity is to combine two AMPs into a hybrid peptide. Hybrid peptides preserve the original amino acid residues from natural AMPs, and some special additional sequences are chosen to enhance antimicrobial activity and reduce hemolytic activity compared to natural AMPs. Currently, hybrid AMPs have been developed by combining several amino acid residue sequences from different AMPs. One of the first hybrid AMP is the hybridization of cecropin A (CA) and melittin (ME). These CA-ME hybrids are more potent than either the parental CA or ME against bacterial strains including methicillin-resistant *Staphylococcus aureus* (MRSA) with reduced hemolysis^4,5^. Hybrid peptides, LfcinB-PG and CA-PG derived from three AMPs, protegrin-1 (PG), bovine lactoferricin (LfcinB) and CA exhibited enhanced cell selectivity and potent anti-inflammatory activity^6^. In addition, the hybrid peptides, LI, LN, and LC designed by combining the typical fragment of human cathelicidin LL-37 with either indolicidin, pig nematode cecropin P1 (CP-1) or rat neutrophil peptide-1 (NP-1) displayed higher antimicrobial activity and lower hemolytic activity than its parental peptides^7^.

LfcinB is a 25-residue multi-functional cationic AMP produced by pepsin digestion from the N-terminal region of bovine lactoferrin (LF), an 80 kDa iron-binding glycoprotein^8^. LfcinB6 (RRWQWR-NH_2_) is the antimicrobial core sequence of LfcinB^9^. It has been reported to be the smallest motif that exhibits antimicrobial activity^10^. KR-12 is the shortest antimicrobial sequence of the human cathelicidin AMP, LL-37^11^. As an analog of KR-12, KR-12-a4 (KRIVKLIKKWLR-NH_2_) is known to exhibit potent antimicrobial activity against gram-positive and gram-negative bacteria and relatively high LPS-neutralization activity^12^. In this study, we designed a hybrid peptide, Lf-KR, composed of LfcinB6 and KR-12-a4, to generate a novel AMP with enhanced cell selectivity for bacterial cells and LPS-neutralization activity along with minimal cytotoxicity. Here, Pro kink was introduced between LfcinB6 and KR-12-a4 to provide cell selectivity. Pro kinks in several α-helical AMPs are known to provide cell selectivity^13–15^. Although Pro is commonly known as a helix-breaking amino acid, it has been found in the putative transmembrane helices of integral membrane proteins^16^. It has been proposed that Pro kink in the membrane-spanning helices facilitates gating by the channel^16^. Also, a number of α-helical membrane-active AMPs including melittin, caerin 1.1, buforin-2, gaegurins, and piscidins contain one or two Pro residues in their central portion^17–20^. One or two Pro residues in these AMPs disturbs the hydrogen bond patterns in the middle of the helix, leading to helix-hinge-helix structures. Introduction of Pro at the central position of some α-helical AMPs increased cell selectivity for bacterial cells than introduction of Gly or Ala^20–22^_._ For this reason, we chosen Pro as the linker in hybrid peptide Lf-KR.

The minimal inhibitory concentration (MIC) against gram-positive and gram-negative bacterial strains was determined to evaluate the antimicrobial activities (including antibiotic-resistant bacteria) of the peptides. Toxicity was assessed by determining hemolytic activity against sheep red blood cells. The effect of monovalent and/or multivalent ions on the antimicrobial activity of the peptides was also investigated. The secondary conformation of the peptides was measured in different aqueous solutions and a membrane mimicking environment by circular dichroism (CD) spectroscopy.

In addition to their direct antimicrobial activity, some AMPs including human cathelicidin LL-37, bovine indolicidin, and defensin are known to suppress lipopolysaccharide (LPS)-induced inflammatory responses by neutralizing and binding LPS^23–26^. Here, the anti-inflammatory activity of the Lf-KR hybrid peptide was established by investigating nitric oxide (NO) and tumor necrosis factor (TNF)‐α cytokine release and their mRNA expression in LPS-stimulated RAW cells.

Biofilms are multicellular aggregates of surface-associated microorganisms that are estimated to cause at least 65% of all infections in humans, particularly implantable device-related infections and chronic disease infections^27,28^. Biofilm-related infections are very difficult to treat in the clinic because of their adaptive resistance to most antibiotics and consequent recalcitrance to treatment with conventional antibiotics. Consequently, there is an urgent need for drugs that effectively treat biofilm-associated infections.

Therefore, we investigated the anti-biofilm activity against multidrug-resistant Pseudomonas aeruginosa (MDRPA) biofilms to evaluate the potential of Lf-KR as a new anti-biofilm agent. The potency of the hybrid peptide to eradicate preformed MDRPA biofilms was evaluated as the minimum biofilm eradication concentration (MBEC). Furthermore, the anti-biofilm activity of Lf-KR on mature MDRPA biofilms was investigated by quantitative analysis of biofilm morphology using a laser confocal scanning microscope (CLSM).

## Results

### Peptide design and characterization

The Lf-KR hybrid peptide was designed so that LfcinB6 and KR-12-a4 were placed at the N-terminus and C-terminus of the peptide, respectively. The N-terminal segment (LfcinB6) and C-terminal segment (KR-12-a4) were connected by Pro. The key physicochemical parameters of the peptides are listed in Table 1. The theoretical molecular weight of each peptide was confirmed by electrospray ionization-mass spectrometry (ESI–MS) (Fig. S1 and Table 1). The calculated and measured values of all peptide weights were consistent, confirming that the Lf-KR peptide was accurately synthesized. HPLC retention time is an important indicator of the relative hydrophobicity of the peptides. The HPLC retention times of LfcinB6, KR-12-a4, and Lf-KR were 13.056, 18.482, and 23.990 min, respectively (Fig. S2), indicating the following hydrophobic order: Lf-KR > KR-12-a4 > LfcinB6.

**Table 1:** Amino acid sequence and physicochemical properties of LfcinB6, KR-12-a4 and Lf-KR.

Peptides	Amino acid sequence	Net charge	t_R_ (min)^a^	μH	Average Mass (Da)	MS Analysis^b^		
						Z	m/z calculated	m/z found
LfcinB6	RRWQWR-NH_2_	+ 3	13.056	−	987.13	[M+H]^+^	988.13	987.0
						[M+2H]^2+^	494.6	493.9
KR-12-a4	KRIVKLIKKWLR-NH_2_	+ 6	18.482	0.912	1581.06	[M+H]^+^	1582.06	1580.5
						[M+2H]^2+^	791.50	791.3
						[M+3H]^3+^	528.0	527.7
Lf-KR	RRWQWRPKRIVKLIKKWLR-NH_2_	+ 9	23.990	0.646	2647.28	[M+2H]^2+^	1324.6	1324.3
						[M+3H]^3+^	883.4	883.4
						[M+4H]^4+^	662.8	662.8

^a^Retention times (t_R_) were determined by analytical RP-HPLC on a C_18_ column (5 mm; 4.6 mm × 250 mm; Vydac) using a gradient of buffer B (0.05% TFA in CH_3_CN/H_2_O 90:10 v/v) in buffer A (0.05%TFA in H_2_O) for 60 min with a flow rate of 1.0 mL/min.

^b^μH: Hydrophobic moment.

^c^Molecular masses were determined by electrospray ionization mass spectrometry (ESI–MS). z: ion charge, m/z: mass-to-charge ratio.

### The tertiary structure of the Lf-KR

**Figure 1:**
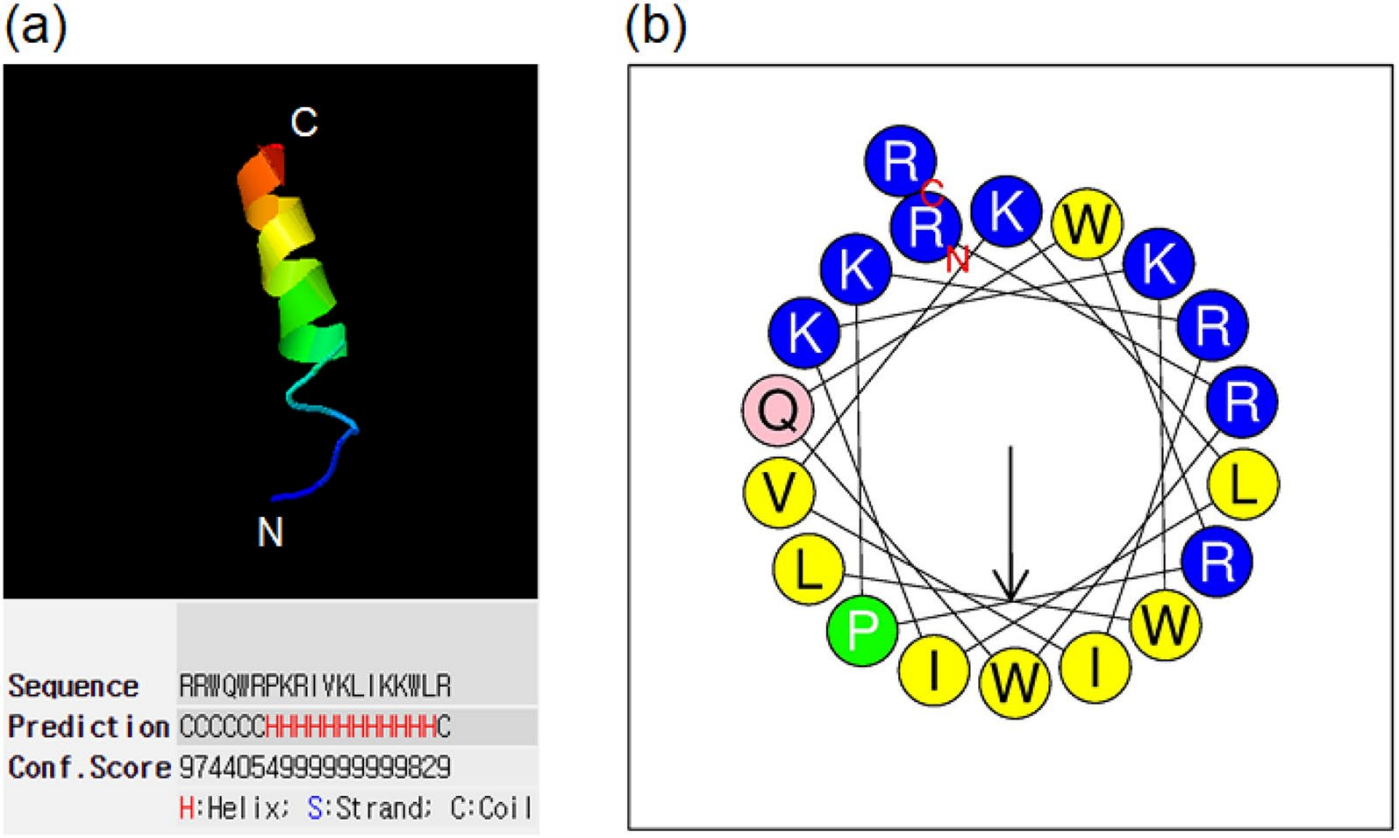
(**a**) Tertiary structure and (**b**) α-helical wheel plot of hybrid peptide Lf-KR predicted by automated I-TASSER server (http://zhanglab.ccmb.med.umich.edu/I-TASSER/) and HeliQuest server (https://heliquest.ipmc.cnrs.fr/cgi-bin/ComputParams.py), respectively. In the α-helical-wheel plot, residues marked in blue and yellow represent positively charged amino acids and hydrophobic amino acids, respectively.

Three predictive models of the three-dimensional structure of Lf-KR were obtained from the I-TASSER server (http://zhanglab.ccmb.med.umich.edu/I-TASSER/)^29^. Model 1 for Lf-KR with the highest C-score was selected (Fig. 1a). The estimated accuracy of Model 1 for Lf-KR was as follows: C score: − 0.27; template modeling TM-score: 0.68 ± 0.12; and root-mean-square deviation (RMSD): 1.4 ± 2.4 Å. The confidence (C)-score estimates the quality of the ITASSER predicted models, typically in the range from − 5 to 2, where a higher value signifies a model with high confidence. The TM-score is a recently proposed scale that measures the similarity between two structures. A TM-score > 0.5 and < 0.17 indicates a model of correct topology and random similarity, respectively. RMSD is the average distance of all the residue pairs in the two structures. As shown in Fig. 1a, Lf-KR adopts a typical α-helical structure at the C-terminus.

### α-helical wheel plot of Lf-KR

The α-helical wheel plot of Lf-KR was predicted using the online analysis tool, HeliQuest server (https://heliquest.ipmc.cnrs.fr/cgi-bin/ComputParams.py) (Fig. 1b). The results showed that Lf-KR carries a + 9 net charge with hydrophobicity of 0.340 and a hydrophobic moment of 0.646 (Table 1). It also has a hydrophobic face consisting of WIWIPLV (Fig. 1b).

### CD spectroscopy

**Figure 2:**
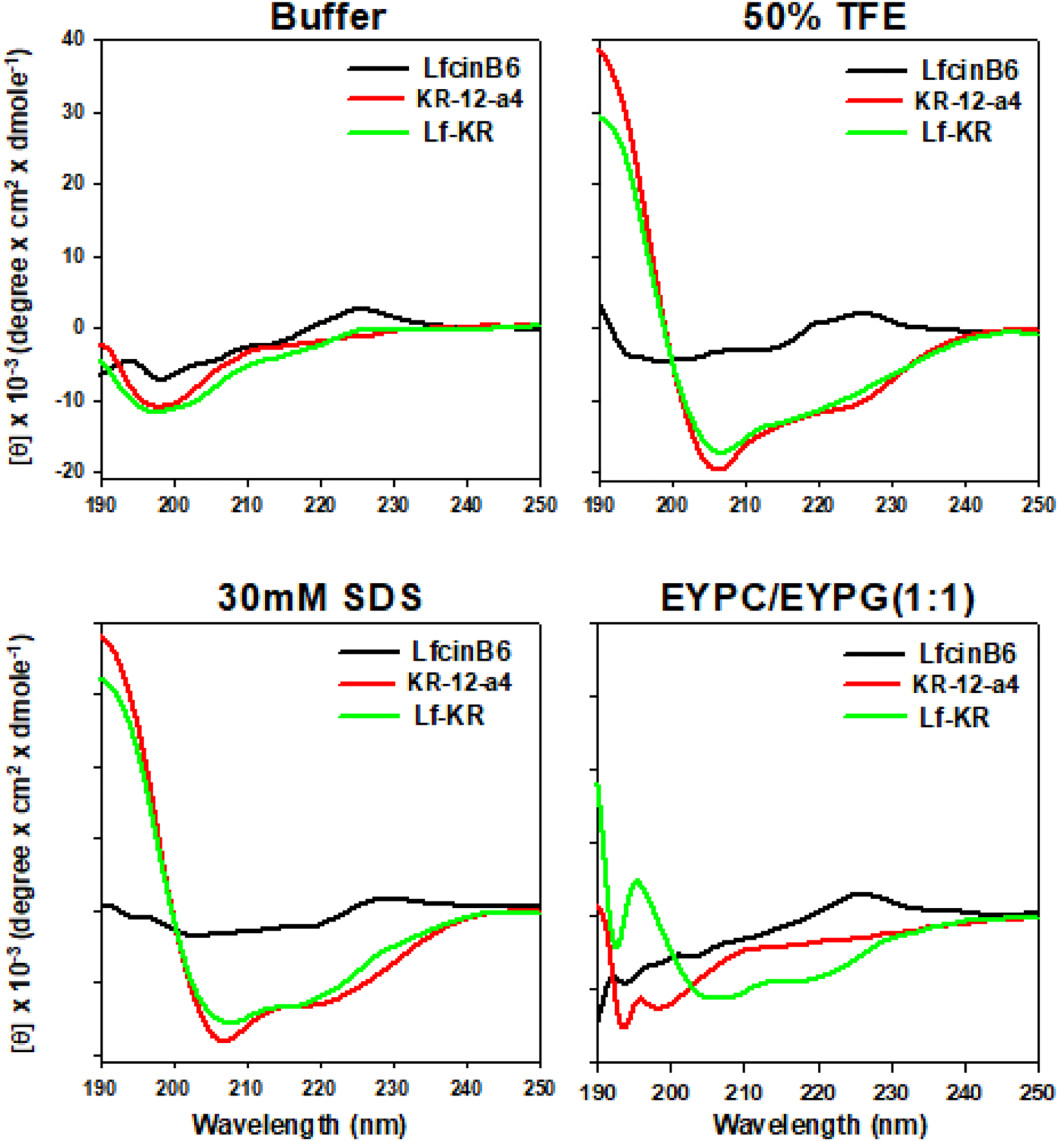
CD (circular dichroism) spectra of LfcinB6, KR-12-a5, and Lf-KR. The mean residue ellipticity was plotted against wavelength. The values from three scans were averaged per sample.

To examine further the secondary structure of the peptides in different environments, CD spectra were measured in mimicking the aqueous environment (10 mM PBS) and in bacterial membrane mimicking environment (50% TFE, 30 mM SDS micelles and EYPC/EYPG (1:1) vesicles). As shown in Fig. 2, except LfcinB6, KR-12-a5, and Lf-KR had a stable α-helical conformation in 50% TFE and 30 mM SDS. They had negative peaks at 208 nm and 222 nm, and a positive peak at 192 nm characteristic of a α-helical structure. In the presence of EYPC/EYPG (1:1) vesicles, only Lf-KR showed an α-helical structure. However, the spectra of all peptides in 10 mM PBS are characteristic of unordered conformations.

### Antimicrobial and hemolytic activities

The antimicrobial activities of the peptides against gram-negative and gram-positive bacteria including antibiotic-resistant bacteria were evaluated by measuring their MICs (Table 2). Except for LfcinB6, all peptides exhibited broad-spectrum antimicrobial activities against a panel of bacteria tested, with MICs ranging from 1 to 64 μM. The geometric mean (GM) of the MIC value of Lf-KR was approximately 6-times lower than that of KR-12-a4. The GM of Lf-KR showed potent antimicrobial activity comparable to melittin that is known to have relatively strong antimicrobial activity among various AMPs. The hemolytic activities of the peptides against sheep red blood cells (sRBCs) were measured to determine their toxicity to mammalian cells (Table 2). All peptides had negligible hemolytic activity even at the highest concentration of 128 μM.

**Table 2:** MIC, MHC and TI of LfcinB6, KR-12-a4 and Lf-KR against different bacterial strains.

Bacterial strains	MIC^a^ (μM)			
	LfcinB6	KR-12-a4	Lf-KR	Melittin
**Gram-positive bacteria**				
*S. aureus* (KCTC 1621)	> 64	16	4	4
*S. epidermidis* (KCTC 1917)	> 64	16	8	4
*B. subtilis* (KCTC 3068)	> 64	4	4	4
**Resistant Gram (+) bacteria**				
MRSA^e^ (CCARM 3089)	> 64	64	8	4
MRSA (CCARM 3090)	> 64	> 64	8	4
MRSA (CCARM 3095)	> 64	> 64	8	8
VREF^f^ (ATCC 51,559)	> 64	64	8	4
**Gram-negative bacteria**				
*E. coli* (KCTC 1682)	> 64	16	8	4
*P. aeruginosa* (KCTC 1637)	> 64	8	8	16
*S. typhimurium* (KCTC 1926)	> 64	8	4	4
**Resistant Gram (−) bacteria**				
MDRPA^g^ (CCARM 2095)	> 64	4	8	8
MDRPA (CCARM 2109)	> 64	64	8	8
GM^b^ (μM)	> 64	43.3	7.0	6.0
MHC^c^ (μM)	> 128	> 128	> 128	5.0
TI^d^ (MHC/GM)	0.5	5.9	36.6	0.8

^a^Minimum inhibitory concentrations (MICs) were determined as the lowest concentration of the peptides that inhibited bacterial growth.

^b^The geometric mean (GM) of the peptide MICs against the tested bacterial strains was calculated. When no antimicrobial activity was observed at 64 μM, a value of 128 μM was used to calculate the therapeutic index.

^c^MHC is the minimum hemolytic concentration that causes 10% hemolysis of sheep red blood cells (sRBCs). When no detectable hemolytic.

activity was observed at 128 μM, a value of 256 μM was used to calculate the therapeutic index.

^d^Therapeutic index (TI) is the ratio of MHC to GM. Larger values indicate greater cell selectivity.

^e^MRSA: methicillin-resistant *Staphylococcus aureus.*

^f^VREF: vancomycin-resistant *Enterococcus faecium.*

^g^MDRPA: multidrug-resistant *Pseudomonas aeruginosa.*

### Cell selectivity (therapeutic index)

The therapeutic index (TI) is defined as the ratio of the minimum hemolytic concentration (MHC) of peptides to the geometric mean (GM) of the peptides (Table 2). TI is an important index for evaluating the clinical application value of antimicrobial agents. Lf-KR had a relatively high TI value of 36.6, approximately sixfold higher than that of KR-12-a4. This result showed that Lf-KR had greater cell selectivity toward bacterial cells than mammalian cells due to its potent antimicrobial activity and minimal hemolytic activity, indicating that it has greater therapeutic potential. In contrast, as a control peptide, melittin (TI = 0.8) has a very low TI value because of its extremely high hemolytic activity.

### Salt insensitivity

The antimicrobial activities of the peptides were tested following the addition of physiological concentrations of different salts for the sensitivity assay. As indicated in Table 3, Lf-KR retained its antimicrobial activity even at high salt concentrations. However, in the presence of high salt concentrations, KR-12-a4 showed significant antimicrobial activity.

**Table 3:** Minimum inhibitory concentration (MIC) values of LfcinB6, KR-12-a4 and Lf-KR in the presence of physiological salts against *E. coli* and *S. aureus.*

Peptides	Peptide alone	150 mM NaCl	4.5 mM KCl	6 μM NH_4_Cl	1 mM MgCl_2_	2.5 mM CaCl_2_	4 μM FeCl_3_
**MIC (μM) against *E. coli* (KCTC 1682)**							
LfcinB6	> 64	> 64	> 64	> 64	> 64	> 64	> 64
KR-12-a4	16	32	16	32	32	32	32
Lf-KR	8	16	8	8	16	16	8
**MIC (μM) against *S. aureus* (KCTC 1621)**							
LfcinB6	> 64	> 64	> 64	> 64	> 64	> 64	> 64
KR-12-a4	16	32	> 32	> 32	> 32	> 32	> 32
Lf-KR	4	4	8	8	8	8	8

### Cytoplasmic membrane depolarization

**Figure 3:**
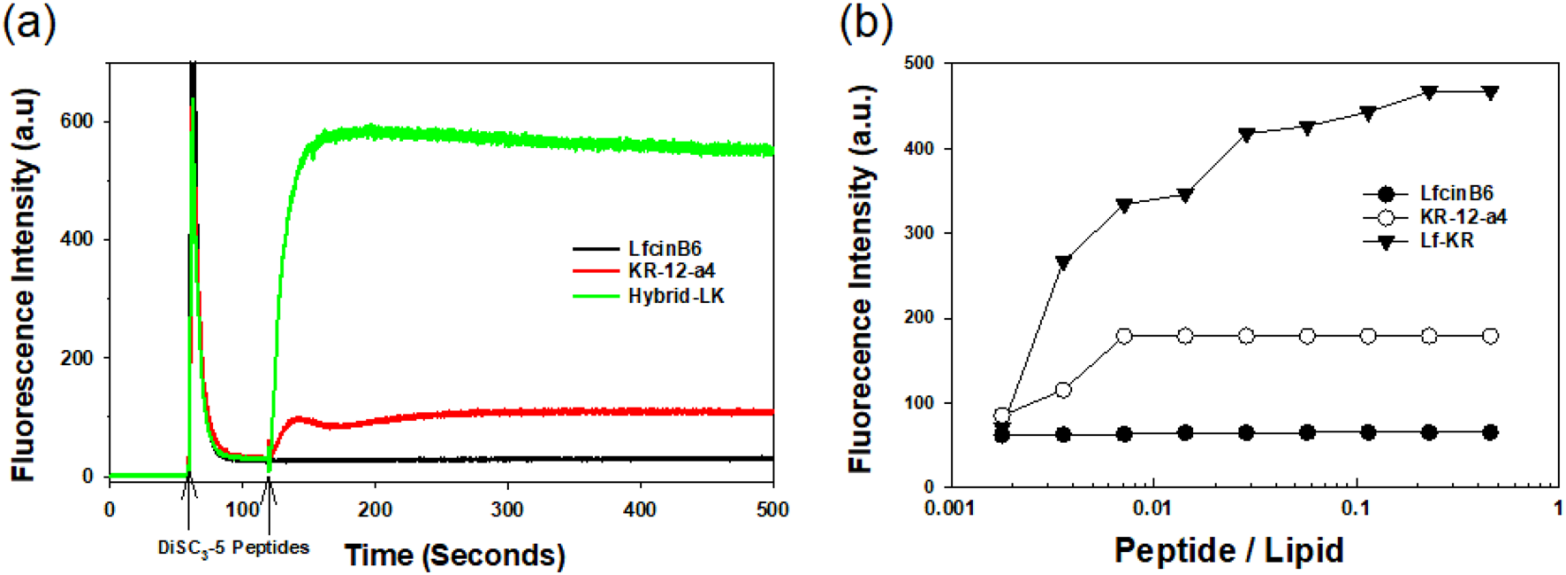
(**a**) Time-dependent cytoplasmic membrane depolarization of *Staphylococcus aureus* (KCTC 1621) treated with the peptides (2 × MIC) assessed by the release of the membrane potential‐sensitive dye, DiSC_3_-5. (**b**) Membrane permeabilization caused by the peptides. Dose-dependent calcein release from EYPE/EYPG (7:3) LUVs induced by LfcinB6, KR-12-a5, and Lf-KR. The fluorescence intensity was measured using an excitation wavelength of 490 nm and an emission wavelength of 520 nm.

A membrane potential-dependent probe (DiSC_3_-5) was used to evaluate the ability of the peptides to depolarize the bacterial cytoplasmic membrane. When the cytoplasmic membrane is permeable and destroyed, the membrane potential is eliminated and DiSC_3_-5 is released into the medium, causing an increase in fluorescence^30^. The membrane depolarization induced by the peptides was monitored for 500 s. LfcinB6 did not induce cell membrane depolarization at 2 × MIC (Fig. 3a). Compared with KR-12-a4, Lf-KR showed faster and stronger membrane depolarization (Fig. 3a).

### Dye leakage

As shown in Fig. 3b, LfcinB6 did not induce any dye leakage from the bacterial membrane mimicking EYPE/EYPG (7:3, v/v) liposomes even at 32 μM. KR-12-a4 reached the maximum dye leakage at 0.5 μM, and no further dye leakage was observed even at 32 μM. However, Lf-KR increased dye leakage in a dose-dependent manner.

### RAW264.7 cell viability

**Figure 4:**
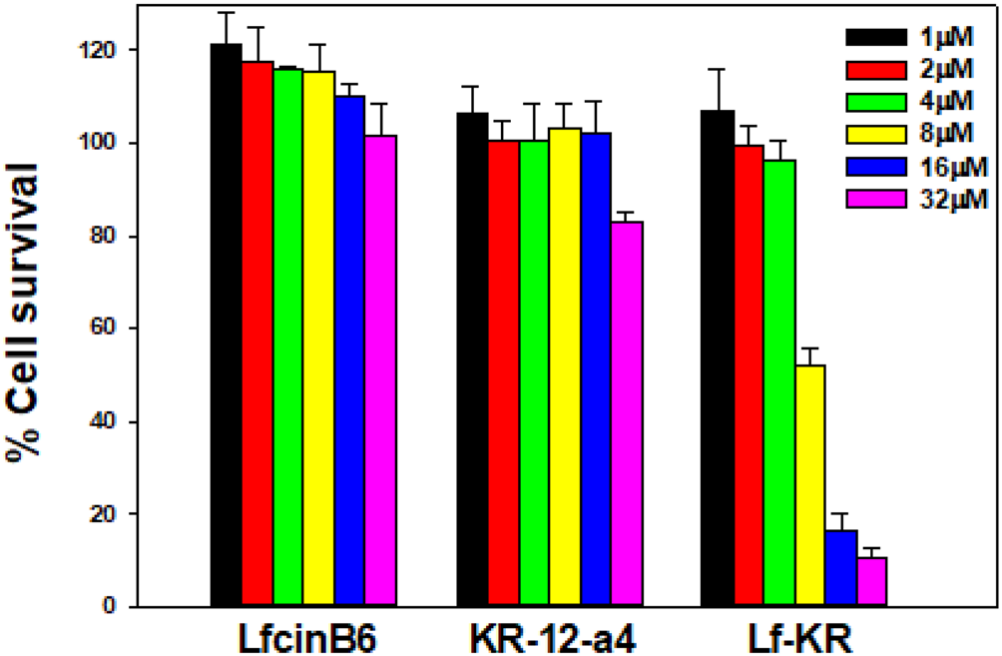
Cytotoxicity of LfcinB6, KR-12-a5, and Lf-KR against mouse macrophage RAW264.7 cells.

Before investigating the inhibitory activity against LPS-induced inflammation, the cytotoxicity of the peptides against RAW264.7 was evaluated using the MTT assay. As illustrated in Fig. 4, none of the peptides affected the viability of RAW264.7 cells at concentrations as high as 4 μM. Therefore, assays examining the effects of the peptides on LPS-induced NO and TNF-α production were conducted at a concentration of 4 μM.

### Inhibition effect of the peptides on LPS-induced NO and pro-inflammatory cytokine TNF-α production

**Figure 5:**
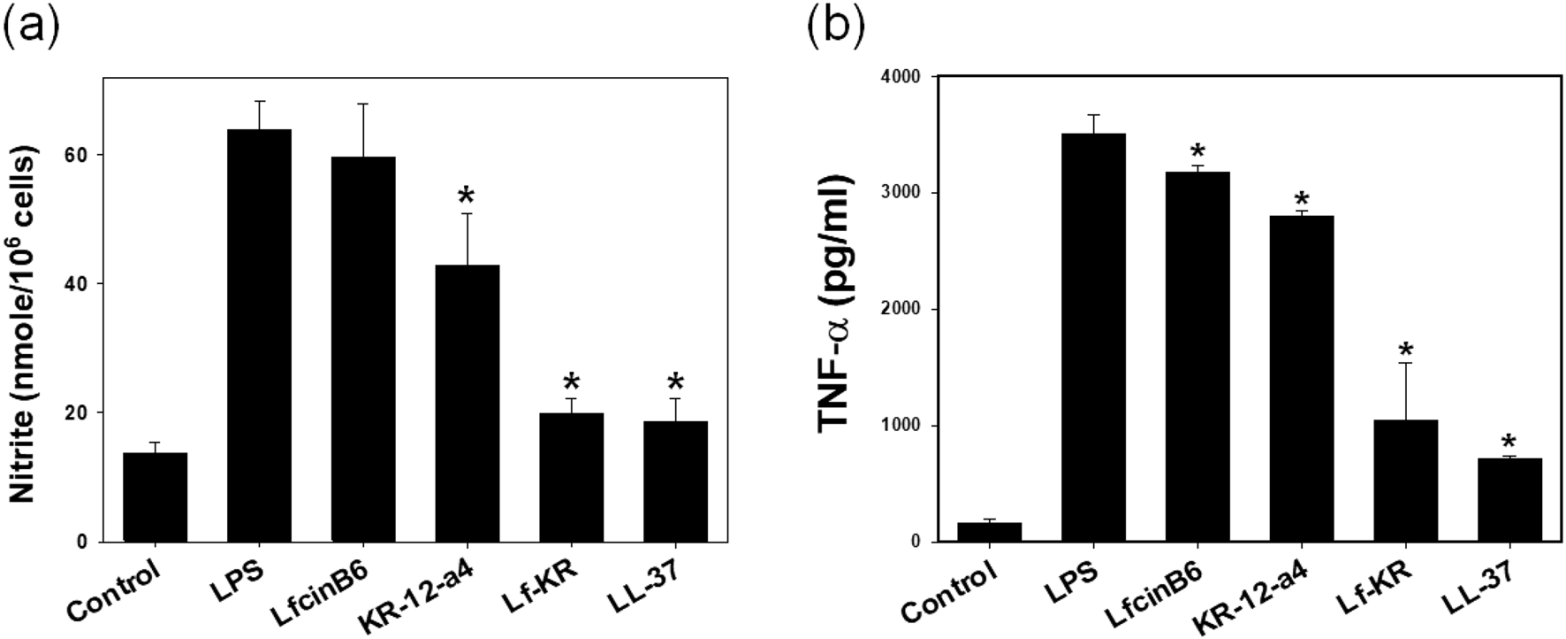
(**a**) Effects of LfcinB6, KR-12-a5, Lf-KR, and LL‐37 on nitric oxide (NO) production in LPS-stimulated RAW264.7 cells. (**b**) Effects of LfcinB6, KR-12-a5, and Lf-KR on TNF-α release from LPS-stimulated RAW264.7 cells. All data represent at least three independent experiments and are expressed as mean ± standard error of the mean (SEM). Data were analyzed by one-way ANOVA with Bonferroni's post-test. Asterisks indicate statistically significant differences (*P < 0.001 for each agonist). Results were similar when the experiments were repeated using different cells. Peptide concentration is 4 μM.

**Figure 6:**
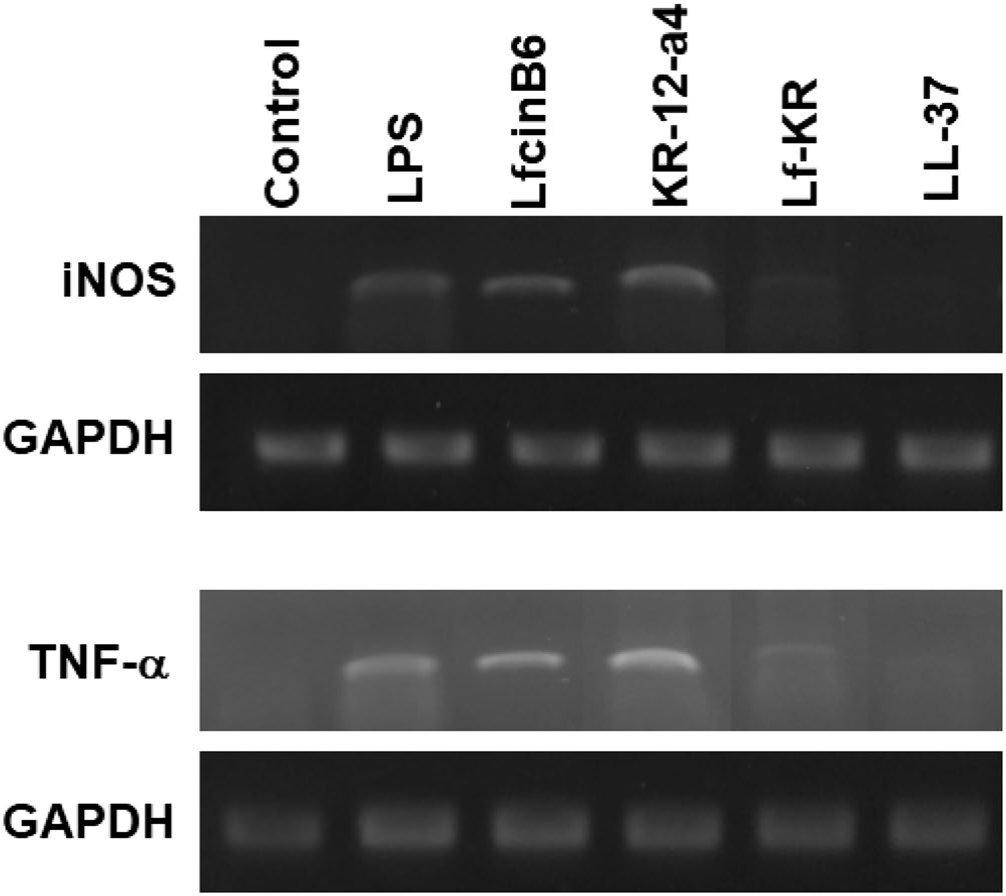
Effects of LfcinB6, KR-12-a5, Lf-KR, and LL‐37 on the mRNA levels of iNOS and TNF-α in LPS-stimulated RAW264.7 cells. RAW264.7 cells (5 × 10^5^ cells/well) were incubated with the peptides in the presence of LPS (20 ng/mL) for 3 h (for TNF-α) or 6 h (for iNOS). Total RNA was isolated and analyzed to determine the levels of iNOS and TNF-α mRNAs by RT-PCR.

LPS, also termed endotoxin, is the main component of the outer membrane of gram-negative bacteria^31^. LPS is an effective agonist of Toll-like receptor 4 (TLR4) on the membrane of many types of immune cells. LPS can induce the release of pro-inflammatory mediators such as nitric oxide (NO) and TNF-α in macrophage cells^32^. The overproduction of pro-inflammatory mediators has been implicated in the pathogenesis of septic shock causing tissue damage and myocardial depression^32–34^. The anti-inflammatory effects of the peptides were evaluated by determining the release of NO and TNF-α from LPS-stimulated RAW264.7 cells (Fig. 5). RAW264.7 cells were stimulated with 200 ng/mL LPS in the presence of 4 μM peptide. NO production was determined by the Griess method that detects nitrite ion (NO_2_^−^) accumulation in the culture medium (Fig. 5a). The inhibitory effect of the peptides on the release of TNF-α in LPS-stimulated RAW264.7 cells was also investigated using commercially available ELISA kits (Fig. 5b). Compared with LfcinB6 and KR-12-a4, the addition of Lf-KR was more effective at inhibiting NO and TNF-α production. Similar to LL-37, a powerful anti-inflammatory peptide, Lf-KR inhibited both NO and TNF-α production in LPS-stimulated RAW264.7 cells at a concentration of 4 μM (Fig. 5). The mRNA expression levels of iNOS and TNF-α were determined by RT-PCR. Lf-KR was as effective as LL-37 in suppressing iNOS (inducible nitric oxide synthase) and TNF‐α expression (Fig. 6 and Fig. S3). These data are in good agreement with the observed inhibition of the NO and TNF-α release by the peptides.

### LPS‐binding activity

**Figure 7:**
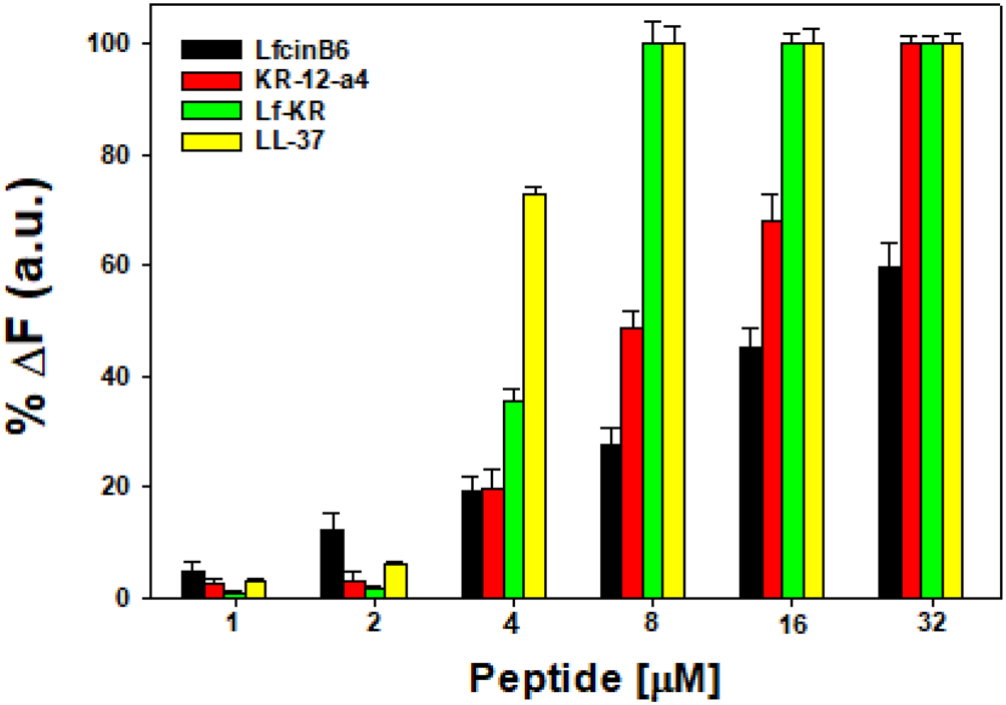
The binding ability of LfcinB6, KR-12-a5, Lf-KR, and LL‐37 to LPS from E. coli 0111:B4. The fluorescence intensity was monitored at an excitation wavelength of 580 nm and an emission wavelength of 620 nm.

We assessed the direct LPS‐binding ability of the peptides using a fluorescence-based displacement assay with BODIPY TR cadaverine (BC). Initially, BC fluorescence is quenched when it binds to free LPS. The introduction of antiendotoxin compounds displaces BC, its fluorescence increases, indicating successful binding of the compound with LPS. As shown in Fig. 7, Lf-KR showed a concentration-dependent increase in BC fluorescence intensity, similar to that of LL‐37, with almost 100% binding to LPS at 8 μM.

### Anti-biofilm activity

**Figure 8:**
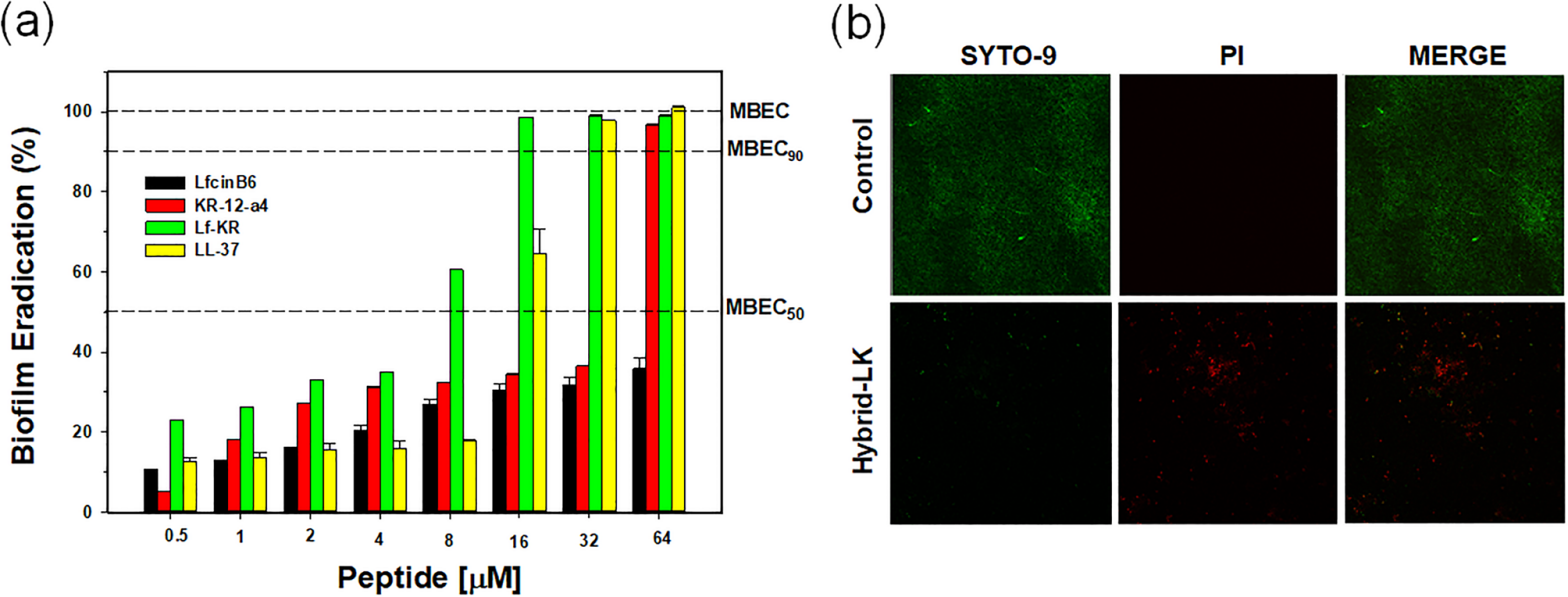
(**a**) Biofilm eradication activity of LfcinB6, KR-12-a5, Lf-KR and LL-37 against multidrug-resistant *Pseudomonas aeruginosa* (MDRPA). The dotted lines indicate 50% and 90% eradication concentrations. (**b**) Effects of Lf-KR on MDRPA mature biofilms were assessed using confocal laser scanning microscopy (CLSM). MDRPA were incubated alone or with Lf-KR (16 μΜ). Biofilms were visualized with live–dead viability staining (SYTO 9/PI). The viable cells exhibited green fluorescence (SYTO 9), whereas the dead cells exhibited red fluorescence (PI).

The anti-biofilm activity of the peptides was determined by their ability to eradicate preformed biofilm cells. We used the minimum biofilm eradication concentration (MBEC) to evaluate the anti-biofilm activity of the peptides. As shown in Fig. 8a and Table 5, Lf-KR showed excellent eradication activity with MBEC_50_ and MBEC against the established MDRPA biofilm at concentrations of 8 μM and 32 μM, respectively. In contrast, the MBECs of KR-12-a4 and LfcinB6 were 64 μM and > 64 μM, respectively. Lf-KR exhibited anti-biofilm activity similar to or slightly higher than that of LL-37, known as an anti-biofilm agent (Fig. 8a and Table 5). To demonstrate the anti-biofilm activity of Lf-KR on mature MDRPA biofilms, a laser confocal scanning microscope (CLSM) was used to visualize the preformed MDRPA biofilm after treatment with Lf-KR. The biofilm was grown for 24 h and treated with Lf-KR for 1 h followed by staining with the fluorescent dyes SYTO 9 (live) and propidium iodide (PI) (dead) bacteria. Under CLSM, a large number of biofilm bacteria were seen aggregated into the control group, mainly living bacteria with green fluorescence (Fig. 8b). The number of MDRPA biofilm cells decreased significantly after treatment with Lf-KR at MBEC (32 μM) for 24 h. The number of dead cells increased, and only scattered bacterial aggregates were present in the field of view (Fig. 8b). The CLSM data showed that Lf-KR had a strong damaging effect on the MDRPA strain biofilm. Collectively, our results suggest that Lf-KR has potential as an anti-biofilm agent for the treatment of MDRPA infection.

## Discussion

Hybridization of two AMPs is an effective way to increase antimicrobial activity or decrease the cytotoxicity of the parental AMPs^35–37^. In this study, a novel hybrid AMP, Lf-KR, was designed by combining the antimicrobial core sequence (LfcinB6) of LfcinB (bovine lactoferricin) and a potent analog (KR-12-a4) of the shortest antimicrobial sequence (KR-12) of human AMP LL-37 and placing a Pro molecular hinge between these two sequences (Table 1).

Consequently, Lf-KR showed remarkably increased antimicrobial activity and cell selectivity against gram-positive and gram-negative bacteria including antibiotic-resistant bacteria compared to LfcinB6 and KR-12-a4, without increasing hemolytic activity (Table 2). It is known that the antimicrobial activity of α-helical amphipathic AMPs is mainly influenced by positive charges and hydrophobicity^38–40^. Previous studies demonstrated that a proper positive charge (usually + 6 to + 7) was essential for antimicrobial activity, but antimicrobial activity did not increase when the positive charge of AMPs was beyond the threshold^41,42^. In this study, the positive charge of Lf-KR was + 9 (Table 1). The HPLC-retention time (RT) typically reflects the hydrophobicity of peptides. Lf-KR (RT: 23.990 min) displayed higher hydrophobicity than KR-12-a4 (RT: 18.482 min) (Table 1 and Fig. S2). Therefore, it is suggested that the enhanced antimicrobial activity of Lf-KR is due to its increased hydrophobicity rather than its increased positive charge.

The therapeutic applications of AMPs lie in their ability to effectively kill bacterial cells without exhibiting significant hemolytic activity. The cytotoxic property is usually conveyed by the therapeutic index, the ratio of the MHC value to the GM value. A high therapeutic index is an indication of two characteristics of the peptides: a high MHC (low hemolysis) and a low MIC (high antimicrobial activity). The therapeutic index of Lf-KR (TI = 36.6) was approximately six times higher than that of KR-12-a4 (TI = 5.9), known to have a relatively high TI value^12^.

The secondary structure of Lf-KR predicted by the Heliquest website is a typical α-helical amphipathic molecule divided into two different sides. One side is the hydrophobic surface composed of hydrophobic amino acid residues such as Ile, Val, Ile, and Trp. The other side is the hydrophilic surface composed of positively charged amino acid residues such as Lys and Arg. CD spectroscopy further confirmed the predicted α-helix structure in membrane-like environments such as TFE and SDS micelles (Fig. 2). Despite its slightly lower α-helix content, Lf-KR showed higher antimicrobial activity than KR-12-a4 (Tables 2, 4). Consistent with the findings of other groups^34,43^, this result indicates that a higher α-helical content of the peptide is not necessarily accompanied by higher antimicrobial activity.

**Table 4:** Mean residual ellipticity at 222 nm ([θ]_222_) and percent α-helical contents of LfcinB6, KR-12-a4 and Lf-KR in aqueous buffer, 50% TFE, 30 mM SDS and EYPC/EYPG (1:1) vesicles.

Peptide	Buffer		50% TFE		30 mM SDS		EYPC/EYPG (1:1)	
	[θ]_222_	% α-helix	[θ]_222_	% α-helix	[θ]_222_	% α-helix	[θ]_222_	% α-helix
LfcinB6	1446.6	rc	− 1073.2	rc	1071.2	rc	1405.1	rc
KR-12-a4	− 1619.9	rc	− 12,483.8	28.74	− 11,431.6	25.55	− 3430.0	rc
Lf-KR	− 1538.6	rc	− 10,967.4	24.14	− 10,534.3	22.83	− 7843.2	14.68

% α-helix = − 100 ([θ]_222_ + 3000)/33,000.

*rc* random coil.

Furthermore, the three-dimensional structure of Lf-KR was modeled using the website I-TASSER and displayed using PDB Viewer software. As a result of modeling, it was found that Lf-KR has an N-terminal random coil structure and the typical α-helical structure at the C-terminus (Fig. 1a). These results show that the Lf-KR peptide can form an amphiphilic α-helix structure under suitable membrane conditions, suggesting its antimicrobial activity.

Studies have reported that salts present at physiological concentrations can impair the antimicrobial activity of AMPs^43–45^. These salts are thought to antagonize antimicrobial activity by competing with peptides for membrane binding^43–45^. Specifically, monovalent free ions such as Na^+^ and K^+^ prevent peptide molecules from binding to the membrane via charge screening effects^43–45^. In the present study, the sensitivity of peptides to salts at physiological concentrations was investigated by monitoring the changes in MIC values. Lf-KR retained antimicrobial activity against *E. coli* and *S. aureus* in the presence of Na^+^, K^+^, NH^4+^, Zn^2+^, Ca^2+^, and Fe^3+^ (Table 3). This result suggests that cation valence [monovalent (NH_4_^+^, K^+^, Na^+^), divalent (Zn^2+^, Ca^2+^), and trivalent (Fe^3+^)] had little or no effect on the strength of the antimicrobial activity of Lf-KR. The high net charge (+ 9) presented by Lf-KR was sufficient to neutralize the charge screening effect induced by the addition of salts, resulting in maintained antimicrobial activity. This result agrees with previous reports that peptides with higher net charges are less sensitive to salts^34,46^. Lf-KR contains three tryptophan residues. Also, the salt insensitivity of Lf-KR might be due to the bulky side chain of tryptophan that could enhance the affinity of this AMP for the bacterial membrane and contribute to its strong antimicrobial activity in the presence of salts^46^.

Most AMPs exhibit antimicrobial activity by destroying bacterial cell membranes through mechanisms ranging from permeabilization to depolarization and transient gaps^47,48^. In the present study, cytoplasmic membrane potential and dye leakage assays were performed to investigate the interaction between the peptides and the cell membrane. The cytoplasmic membrane potential assay indicated that Lf-KR possessed the ability to damage the bacterial cytoplasmic membrane (Fig. 3a). In addition, Lf-KR increased dye leakage via the bacterial membrane, mimicked by EYPE/EYPG (7:3, v/v) liposomes, in a dose-dependent manner (Fig. 3b). Therefore, the enhanced antimicrobial activity of Lf-KR is due to increased permeabilization and depolarization of microbial membranes. Membrane destruction leading to loss of barrier function results in leakage of cytoplasmic content and cell death.

LPS, a major component of the outer membrane of gram-negative bacteria, can cause inflammation, sepsis, and shock. Following bacterial death under the action of antimicrobial agents, a large amount of LPS detaches from the cells and enters the blood circulation of the body, activates the inflammatory signaling pathway, and releases inflammatory mediators such as NO and TNF-α^49,50^. Numerous AMPs such as LL-37 and indolicidin-derived peptides^23–26^ have been described that not only show broad-spectrum antimicrobial activity but also inhibit the release of pro-inflammatory cytokines. In the present study, the anti-inflammatory activity of Lf-KR was investigated and compared with that of LL-37. At non-toxic concentrations, Lf-KR significantly inhibited NO and TNF-α production and their gene expression in LPS-stimulated RAW264.7 cells with a potency equivalent to LL-37 (Figs. 5, 6). This result suggests that Lf-KR has the potential to be developed as an anti-inflammatory agent by blocking LPS-mediated inflammatory mediators. The exact mechanism of the anti-inflammatory activity of AMPs in LPS-stimulated RAW264.7 cells is controversial. It is known that one of the possible mechanisms of the anti-inflammatory activity of AMPs is direct interaction with LPS. The BODIPY TR cadaverine (BC) assay demonstrated that the LPS-binding activity of Lf-KR was similar to that of LL-37, known to have powerful LPS-binding activity (Fig. 7). This result suggested that the enhanced anti-inflammatory activity of Lf-KR is due to an increase in the direct interaction with LPS.

Biofilms are considered one of the most resistant mechanisms of bacterial cell survival that cannot be managed by the available conventional antibiotics^27,28^. In general, compared to planktonic cells, biofilms take tenfold to 1000-fold higher concentrations of traditional antibiotics to be eradicated due to their specific nature of resistance including the extracellular lipopolysaccharide matrix that delays antibiotic penetration and hence leads to the loss of treatment efficiency^27,28^. It is encouraging that AMPs have anti-biofilm activity because of their special mode of action^51,52^. In this study, the ability of Lf-KR to eradicate preformed MDRPA biofilms was evaluated in vitro. Notably, Lf-KR eradicated 100% of mature biofilms at a concentration of 16 μM (Fig. 8a and Table 5). Although the mechanism of action of AMPs against biofilms is not clear, one possible explanation is that AMPs have anti-biofilm activity by forming pores within the lipid components of the biofilm, passing through the extracellular biofilms, or dispersing the biofilms^53,54^. Therefore, CLSM was used to examine mature MDRPA biofilm after treatment with the peptides to visualize the possible biofilm-disruptive activity. Lf-KR disrupted mature MDRPA biofilm as assessed by CLSM (Fig. 8b). Collectively, our results suggest that Lf-KR is a potential bacterial biofilm-eradicating agent for the treatment of mature MDRPA biofilms.

**Table 5:** Minimum biofilm eradication concentration (MBEC) of LfcinB6, KR-12-a4, Lf-KR and LL-37.

Peptides	MBEC_50_ (μM)	MBEC_90_ (μM)	MBEC (μM)
LfcinB	> 64	> 64	> 64
KR-12-a4	64	64	64
Lf-KR	8	16	32
LL-37	16	32	32

## Conclusion

In the present study, we developed a novel hybrid AMP, Lf-KR, with enhanced cell selectivity by combining LfcinB6 and KR-12-a4 and introducing a Pro hinge between these two sequences. Lf-KR exerted high cell selectivity for bacterial cells. Lf-KR has broad-spectrum antimicrobial activity against gram-positive and gram-negative bacteria, including several antibiotic-resistant strains, without increasing hemolytic activity. Lf-KR retained its antimicrobial activity in the presence of salts at physiological concentrations. Lf-KR significantly inhibited the expression and production of pro-inflammatory cytokines (NO and TNF-α) in LPS-stimulated mouse macrophage RAW264.7 cells. Lf-KR showed a strong eradication effect on the preformed MDRPA biofilm. It was observed by CLSM that Lf-KR disrupted the structure of the preformed MDRPA biofilm. Collectively, our results suggest that Lf-KR is a putative candidate for the development of antimicrobial, anti-inflammatory, and anti-biofilm agents.

## Materials and methods

### Materials

Egg yolk L-phosphatidylethanolamine (EYPE), egg yolk l-phosphatidyl-DLglycerol (EYPG), LPS purified from *Escherichia coli* O111:B4, were purchased from Sigma-Aldrich (St. Louis, MO, USA). 3,3′-Dipropylthiadicarbocyanine iodide (diSC_3_-5), SYTO 9 and propidium iodide (PI) were supplied from Molecular Probes (Eugene, OR, USA). HyClone Dulbecco's modified Eagle's medium (DMEM) and fetal bovine serum (FBS) were obtained from SeouLin Bioscience (Seoul, Korea). The enzyme‐linked immunosorbent assay (ELISA) kits for TNF‐α were procured from R&D Systems (Minneapolis, MN).

### Bacterial strains

Bacterial strains were chosen to detect the minimal inhibitory concentration (MIC) of the peptides, as previously described^55^. Three strains of gram-positive bacteria (*Bacillus subtilis* [KCTC 3068], *Staphylococcus epidermidis* [KCTC 1917], and *Staphylococcus aureus* [KCTC 1621]) and three strains of gram-negative bacteria (*Escherichia coli* [KCTC 1682], *Pseudomonas aeruginosa* [KCTC 1637], and *Salmonella typhimurium* [KCTC 1926]) were procured from the Korean Collection for Type Cultures (KCTC) of the Korea Research Institute of Bioscience and Biotechnology (KRIBB). Methicillin-resistant *Staphylococcus aureus* (MRSA) [CCARM 3089, CCARM 3090, and CCARM 3095] and multidrug-resistant *Pseudomonas aeruginosa* strains (MDRPA) [CCARM 2095, and CCARM 2109] were obtained from the Culture Collection of Antibiotic-Resistant Microbes (CCARM) of Seoul Women's University in Korea. Vancomycin-resistant *Enterococcus faecium* (VREF) [ATCC 51559] was supplied from the American Type Culture Collection (Manassas, VA, USA).

### Peptide synthesis and characterization

Peptides are synthesized using solid-phase peptide synthesis employing a fluorenylmethoxycarbonyl (Fmoc) protecting group strategy^56^. The peptides were purified by reversed-phase preparative HPLC on a C18 column (250 mm × 20 mm; Vydac) using an appropriate 0–90% H_2_O/CH_3_CN gradient in the presence of 0.05% trifluoroacetic acid. The purity (≥ 95%) and hydrophobicity were analyzed by reversed-phase analytical HPLC on C18 column (4.6 mm × 250 mm; Vydac). The molecular masses of purified peptides were determined by ESI–MS (electrospray ionization-mass spectrometry) (Framingham, MA, USA).

### Bioinformatic analysis

The α-helical wheel plot, net charge and hydrophobic moments were calculated online using the HeliQuest server. The three-dimensional structure of Hybrid-LK was predicted online using I-TASSER server^29^.

### Circular dichroism (CD) spectroscopy

CD studies were done in a JACSO-(J-715) spectropolarimeter (Jasco, Japan) with 0.1 mm quartz cuvette at 25 °C. The peptides were dissolved in 10 mM sodium phosphate buffer (pH 7.4), 50% TFE, and 30 mM SDS micelles. The percentage of α-helix of the peptides was calculated using the following equation, α-helix (%) =  − 100 × (θ_222_ + 3000)/33,000.

### Minimum inhibitory concentration (MIC)

The minimal inhibitory concentrations (MICs) of the peptides against bacterial strains were determined via the broth microbroth dilution protocol recommended by the Clinical and Laboratory Standard Institute (CLSI)^30,34^. In brief, mid-logarithmic phase of bacteria was diluted with Mueller–Hinton broth (MHB) (Difco, USA) and added to a microtiter 96-well plate (2 × 10^6^ CFU/well). A two-fold serial dilution of samples (concentration range: 1–64 μM) was subsequently added, and the plate was incubated for 24 h at 37 °C. The MIC (µg/L) was taken as the lowest concentration of the antimicrobial that inhibited the visible growth of the bacteria. All experiments were performed in triplicate and included growth and sterility controls.

### Hemolytic activity

The hemolytic activity of the peptides was determined as the amount of hemoglobin released by the lysis of sheep red blood cells (sRBCs), as previously described^55^. Briefly, fresh sheep red bloods were washed with PBS and 4% blood solution was prepared in PBS. In a 96-well plate, 100 μl of varying concentrations of peptides were prepared. Another 100 μl of 4% blood solution was added to each well. The plate as then incubated for 1 h at 37 °C. The plate was centrifuged and the OD_450_ of the supernatant was measured. 0.1% triton-X100 was taken a positive control and PBS was taken as a negative control.

### Cytoplasmic membrane depolarization assay

The cytoplasmic membrane depolarization activity of the peptides was determined with the membrane potential-sensitive fluorescent dye, diSC_3_-5, as previously described^43^. Briefly, logarithmic growing *S. aureus* (KCTC 1621) cells were harvested and diluted to OD_600_ = 0.05 in 5 mM HEPES buffer (pH 7.4, containing 20 mM glucose). The cell suspension was further incubated with 0.4 μM diSC_3_-5 and 100 mM K^+^ until no further reduction of fluorescence. The fluorescence was recorded (excitation λ = 622 nm, emission λ = 670 nm) with a Shimadzu RF-5300PC fluorescence spectrophotometer (Kyoto, Japan). Subsequently, 3 ml of cell suspension was added to a 1 cm quartz cuvette and mixed with the peptides at their 2 × MIC. Changes in the fluorescence were recorded from 0 to 500 s.

### Dye leakage assay

Prepared calcein-entrapped large unilamellar vesicles (LUVs) were optimized using a previous method^57^. The negatively charged lipids composed of EYPE/EYPG (7:3, w/w) were dissolved in chloroform, dried with a stream of nitrogen and resuspended in dye buffer solution (70 mM calcein, 10 mM Tris, 150 mM NaCl, and 0.1 mM EDTA, pH 7.4). The suspension was subjected to 10 freeze–thaw cycles in liquid nitrogen and extruded 21 times through a LiposoFast-Extruder (Avestin, Inc., Canada) equipped with filters of 100 nm pore size. Untrapped calcein was removed from the liposome by gel filtration on a Sephadex G-50 column Calcein leakage from liposomes was monitored at room temperature by measuring fluorescence intensity at an excitation wavelength of 490 nm and emission wavelength of 520 nm on a Shimadzu RF-5300PC fluorescence spectrophotometer (Kyoto, Japan). Complete dye release was obtained using 0.1% Triton X-100.

### Cytotoxicity against RAW264.7 macrophage cells

The cytotoxicity of the peptides against mouse macrophage RAW264.7 cells was assessed by MTT assay^58^. Briefly, RAW264.7 cells were cultured in DMEM (Gibco) with 10% FBS in a humidified atmosphere containing 5% CO_2_ at 37 °C. The cells were added to 96-well plates at a final concentration for 2 × 10^4^ cells per well in DMEM and cultured overnight. TZP4 was then added and incubated for 48 h. MTT (50 µL, 0.5 mg/mL) was added to the 96-wellplate and incubated at 37 °C for 4 h. Subsequently, 150 µL of DMSO was added to dissolve the formed formazan crystals after the supernatant was discarded, and the OD was measured using a microplate reader (Bio-Tek Instruments EL800, USA) at 550 nm. Cell viability was expressed as (A_550nm_ of treated sample)/(A_550nm_ of control) 100%.

### Measurement of nitric oxide or tumor necrosis factor‐α release from LPS-stimulated RAW264.7 cells

Peptide-induced inhibition of nitric oxide (NO) and proinflammatory cytokine, tumor necrosis factor (TNF)‐α production in LPS-stimulated macrophage cells were measured as previously described^59^. In brief, RAW264.7 murine macrophage cells (2 × 10^6^ cell/mL) were plated and adhered to a 96 well plates (100 µL/well) and stimulated with LPS from *E. coli* O111:B4 (20 ng/mL) in the presence or absence of peptide for 24 h. After 24 h incubation, the culture supernatant was collected for enzyme linked immunosorbent assay (ELISA) to detect the level of nitric oxide (NO) and inflammatory cytokine TNF-α. The nitrite level was determined using Griess reagent (1% sulfanilamide, 0.1% naphthylethylenediamine dihydrochloride and 2% phosphoric acid). Release of TNF‐α was detected using DuoSet ELISA mouse TNF-α (R&D Systems, Minneapolis, USA) according to the manufacturer’s protocol.

### Reverse-transcription polymerase chain reaction (RT-PCR)

RT-PCR was performed as previously described^55^. RAW264.7 cells were plated into 6-well plates at a concentration of 5 × 10^5^ cells/well and stimulated with *E. coli* O111:B4 LPS (20 ng/mL) in the presence or absence of the peptides. After incubation of 3 h (for TNF-α) and 6 h [for inducible nitric oxide synthase (iNOS)], total RNA was extracted using TRIzol® reagent (Invitrogen) and RNA concentration quantified using Nanodrop spectrophotometer (BioDrop, UK). cDNA was synthesized from 2 µg of total RNA using Oligo-d(T)15 primers and PrimeScript Reverse Transcriptase kit (Takara, Japan) according to the manufacturer’s protocol. The cDNA products were amplified using following primers: iNOS (forward 5ʹ-CTGCAGCACTTGGATCAGGAACCTG-3ʹ, reverse 5ʹ-GGGAGTAGCCTGTGTGCACCTGGAA-3ʹ); TNF-α (forward 5ʹ- CCTGTAGCCCACGTCGTAGC-3ʹ, reverse 5ʹ-TTGACCTCAGCGCTGAGTTG-3´) and GAPDH (forward 5ʹ-GAGTCAACGGATTTGGTCGT-3ʹ, reverse 5ʹ- GACAAGCTTCCCGTTCTCAG-3ʹ). The PCR amplification was carried out at initial denaturation at 94 °C for 5 min, followed by forty cycles of denaturation at 94 °C for 1 min, annealing at 55 °C for 120 s and extension at 72 °C for 1 min, with a final extension at 72 °C for 5 min. The PCR products were separated by electrophoresis and visualized under UV illumination.

### LPS‐binding assay

The LPS‐binding ability of the peptides was determined by a BODIPY‐TRcadaverine (BC) displacement assay^60,61^. Briefly, LPS from *E. coli* O111:B4 (25 μg/ml) was incubated with BC (2.5 μg/ml) and peptide (1‐32 μM) in Tris buffer (50 mM, pH 7.4) for 4 h. A volume of 2 ml of this mixture was added to a quartz cuvette. Fluorescence was recorded at an excitation wavelength of 580 nm and an emission wavelength of 620 nm with a Shimadzu RF‐5301 PC fluorescence spectrophotometer (Shimadzu Scientific Instruments). The percentage fluorescence was calculated using formula: %ΔF (AU) = [(F_obs_ − F_0_)/(F_100_ − F_0_)] × 100, where F_obs_ is the observed fluorescence at a given peptide concentration, F_0_ is the initial fluorescence of BC with LPS in the absence of peptides, and F_100_ is the BC fluorescence with LPS cells upon the addition of 10 μg/ml polymyxin B.

### Biofilm eradication assay (MBEC)

Biofilm eradication, determined as the MBEC of the peptides, was assessed according to methods from the literature using the Calgary Biofilm Device (Innovotech, Edmonton, Canada)^62,63^. Briefly, 1 × 10^6^ CFU/mL of bacteria were suspended in 150 μL of appropriate nutrient media (LB media) and placed in 96-well microtiter plates with peg lids (Innovotech, Edmonton, Canada; product code: 19111) to establish biofilms. Plates were sealed with parafilm and incubated at 37 °C for 24 h in a shaking incubator at 110 rpm. Lids of the plates were then removed, rinsed with 0.01 M PBS, and transferred to sterile 96-well plates containing serial dilutions of the peptides: the final volume with the media was 200 μL/well. Plates were then incubated at 37 °C for 24 h in a shaking incubator at 110 rpm. After 24 h of treatment, the peg lid of each plate was removed, rinsed with buffer, and transferred to a recovery plate containing 200 μL of nutrient media. Recovery plates were thereafter sonicated in a water bath for 10–15 min to dislodge biofilms. Peg lids were removed and plates were incubated overnight (for 24 h) at 37 °C in a shaking incubator at 110 rpm to recover viable bacteria, resulting in turbidity. The MBEC values were recorded as the lowest concentration resulting in eradication of the biofilm (i.e., no turbidity after the final incubation period relative to sterility controls). Experiments were performed in triplicates, and the median value of each experiment was presented.

### Confocal laser scanning fluorescence microscopy (CLSM)

MDRPA (1 × 10^6^ CFU/mL) was cultured in 24-well plates containing discs placed in MHB-glucose medium, for 24 h to form biofilms. Discs with planktonic cells were washed with 1 × PBS thrice and placed in fresh 24-well plates containing Lf-KR (MBEC: 16 μM), and plates were incubated for 6 h. Discs were removed, washed twice with 1 × PBS, and concomitantly stained with 6.7 μM SYTO 9 and 40 μM PI. After incubation in the dark at 37 °C for 30 min, planar images of biofilm mass in the discs were visualized using CLSM (Zeiss LSM 710 Meta, ZEISS Microscopy, Jena, Germany), and analyzed using ZEN 2009 Light Edition software (ZEISS Microscopy, Jena, Germany).

## Supplementary Information

Supplementary Figures.
