# Supplementary material for: A novel hybrid peptide composed of LfcinB6 and KR-12-a4 with enhanced antimicrobial, anti-inflammatory and anti-biofilm activities
Source: Sci Rep. 2022 Mar 14;12:4365. doi: 10.1038/s41598-022-08247-4 (PMC8921290; doi:10.1038/s41598-022-08247-4)

**Supplementary data**

**Fig. S1.** Molecular masses of synthetic peptides (LfcinB6, KR-12-a4 and Lf-KR) were determined by electrospray ionization-mass spectrometry (ESI-MS).

**Fig. S2**. Analytical RP-HPLC profiles of synthetic peptides (LfcinB6, KR-12-a4 and Lf-KR). Peptides were eluted for 60 min with a flow rate of 1.0 mL/min by analytical RP-HPLC on a C_18_ column (5 mm; 4.6 mm × 250 mm; Vydac) using a gradient of buffer B (0.05% TFA in CH_3_CN/H_2_O 90:10 v/v) in buffer A (0.05 %TFA in H_2_O).

**Fig. S3.** Effects of LfcinB6, KR-12-a5, Lf-KR, and LL‐37 on the mRNA levels of iNOS (a)

and TNF-α (b) in LPS-stimulated RAW264.7 cells.

**Fig. S1**

**
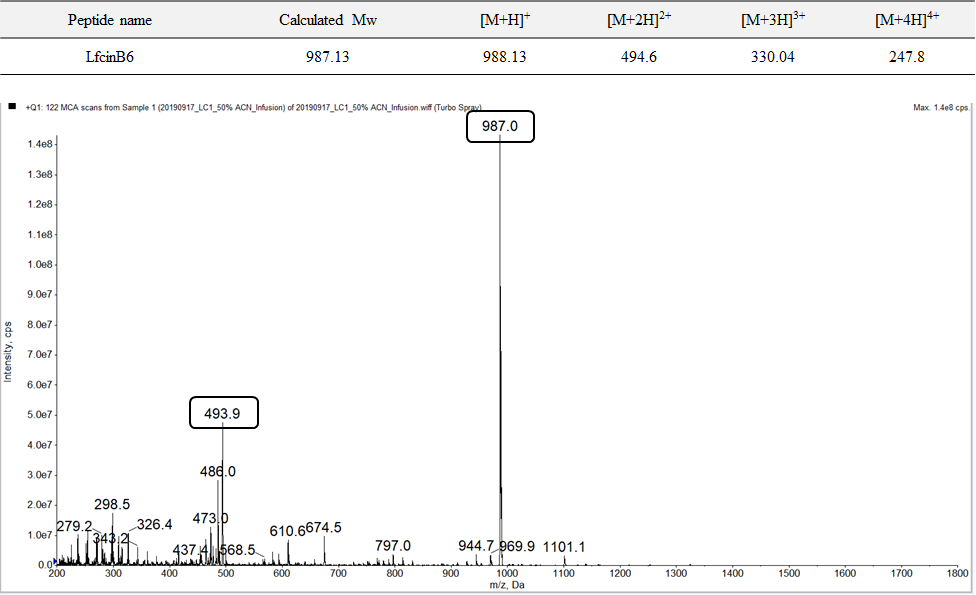
**

**Fig. S1**

**
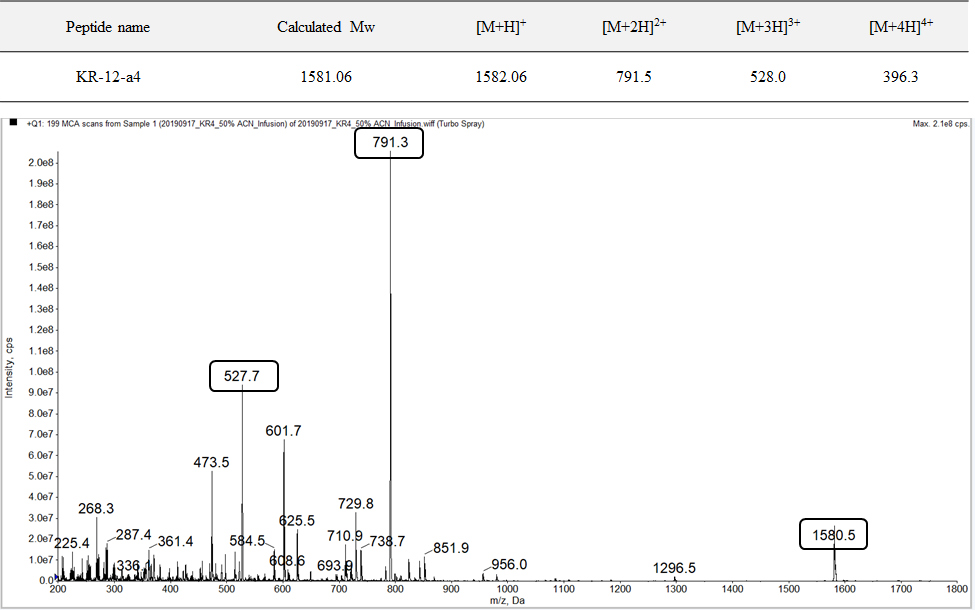
**

**Fig. S1**

**
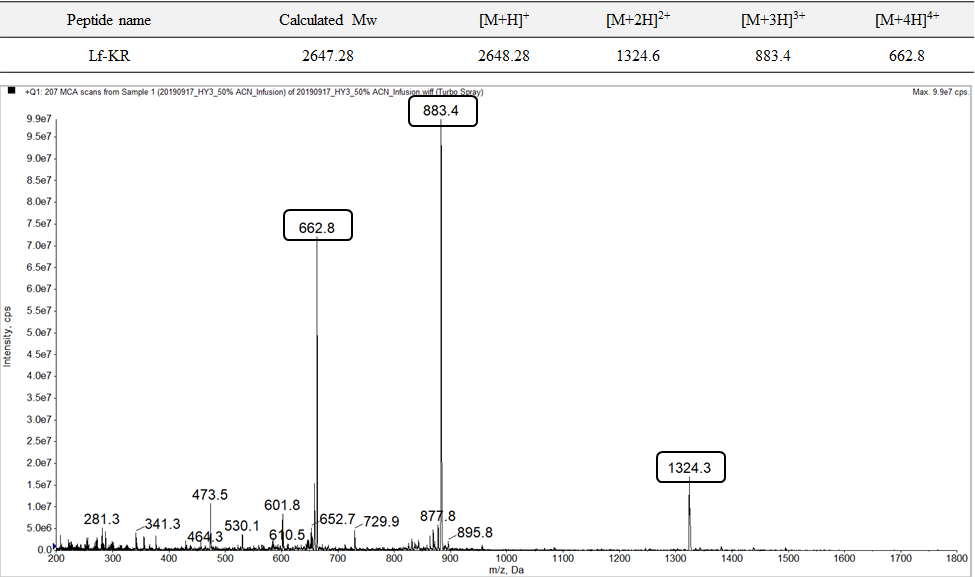
**

**Fig. S2**


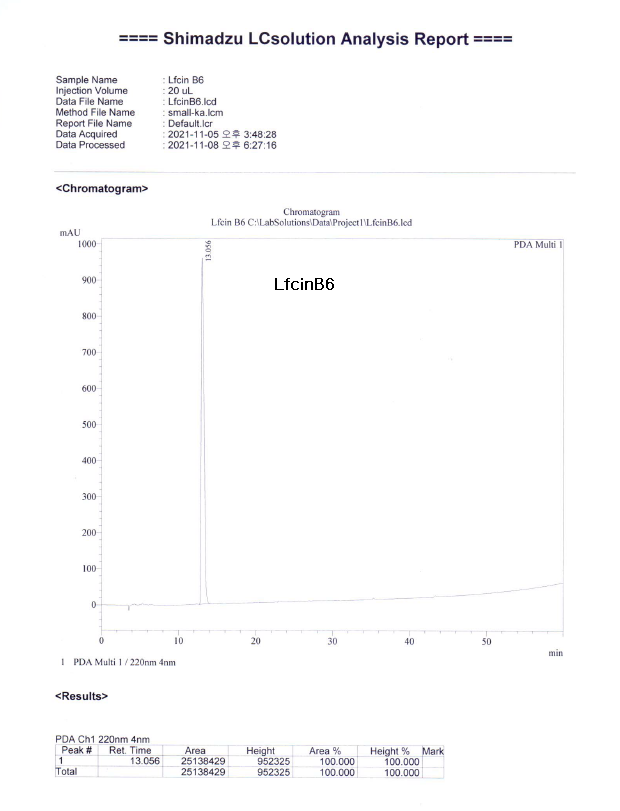


**Fig. S2**


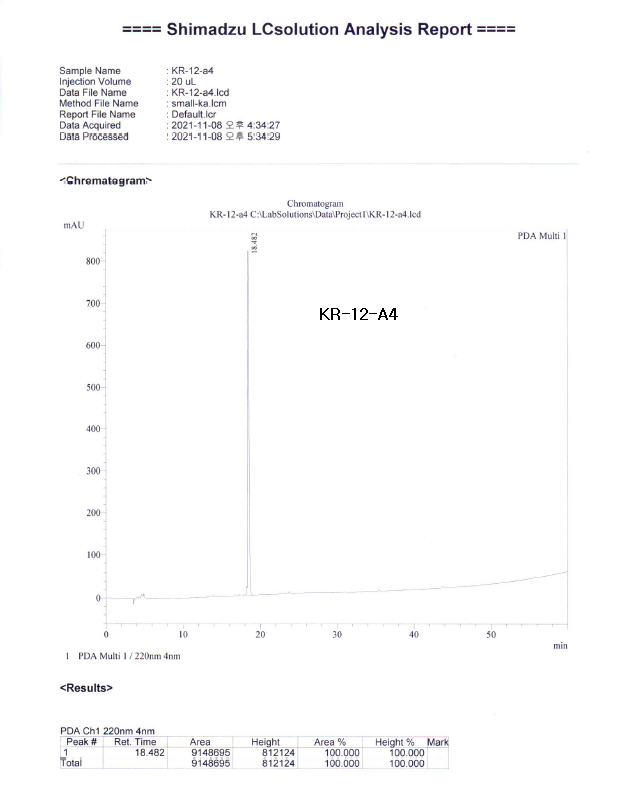


**Fig. S2**


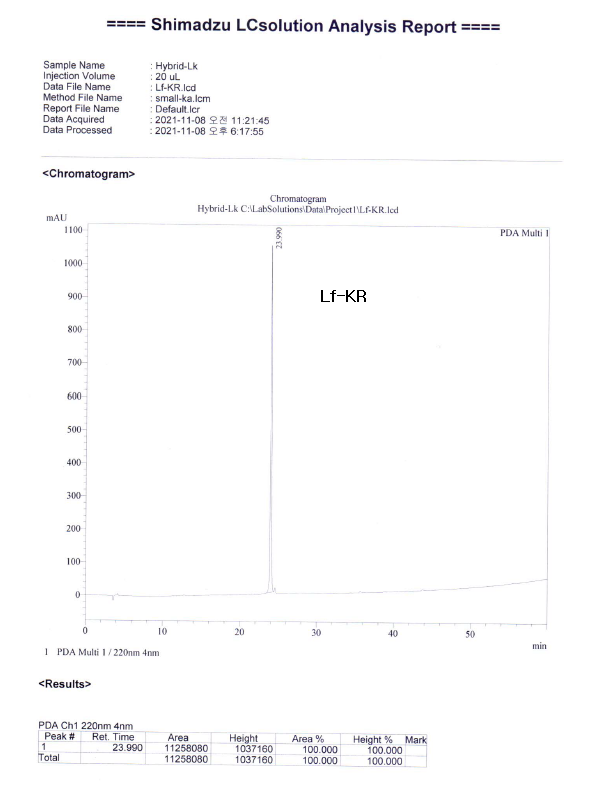


**Fig. S3-a**


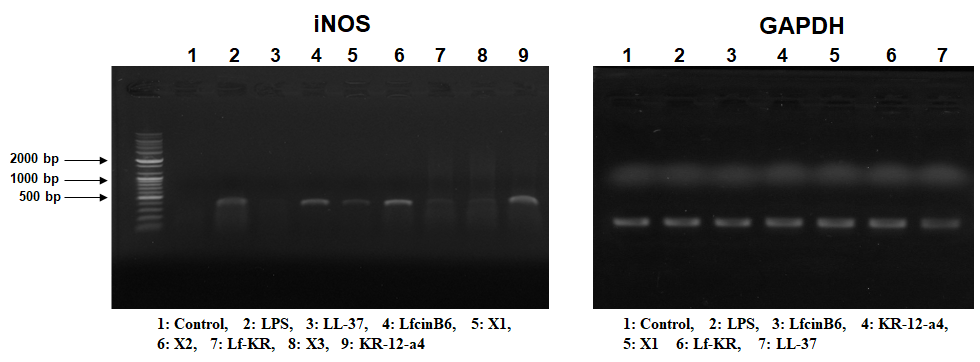


**Fig. S3-b**


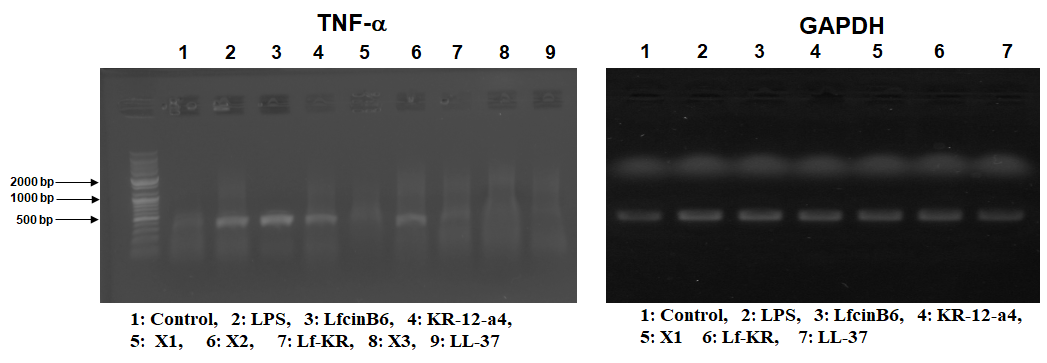

Supplement: Supplementary file 1 — Supplementary Figures. [file 41598_2022_8247_MOESM1_ESM.docx]
